# An investigation into the prevalence of dog bites to primary school children in Trinidad

**DOI:** 10.1186/1471-2458-8-85

**Published:** 2008-03-05

**Authors:** Karla Georges, Abiodun Adesiyun

**Affiliations:** 1 School of Veterinary Medicine, Faculty of Medical Sciences, The University of the West Indies, St Augustine Campus, EWMSC, Trinidad and Tobago

## Abstract

**Background:**

To estimate the prevalence of dog bites to primary school children between the ages of 8–12 years using a semi-structured interview process. With the increase in the pet population and popularity of dangerous breeds of dog and a high stray dog population combined with a dearth of information on the risk of dog attacks to children in Trinidad, a semi-structured interview process was used to determine risk factors associated with dog attacks.

**Methods:**

A questionnaire survey of 1109 primary school children between the ages of 8–12 years was conducted in Trinidad from November 2002 to September 2003. The survey was conducted to determine the risk factors such as age, gender, size of dog and relationship of dog and victim, in dog bite incidents. The chi-square statistic and odds ratios were used to estimate risk factors for a bite incident.

**Results:**

Twenty-eight percent of children were bitten at least once by a dog. Gender (male) and owning a dog were statistically significant risk factors (p = 0.003 and 0.008 respectively, χ^2 ^*df*, 95% confidence). Most attacks occurred outside of the home (58.0%) followed by the victims' home (42.0%) and were by a dog known but not owned (54.6%) by the victim. Many victims (33.0%) were bitten without having any interaction with the dog and the majority (61.9%) of victims did not receive professional medical assistance. Overall, the lower leg or foot was most often injured (39.3%).

**Conclusion:**

A public educational campaign is needed on responsible pet ownership. In addition, children must be taught effective ways of avoiding attacks or reducing injury in the event of a dog attack. The Dangerous dogs Act 2000 must be proclaimed in parliament by the Government of Trinidad and Tobago to exert more pressure on pet owners to safeguard the public from the menace of dog attacks.

## Background

Trinidad and Tobago is a two Island Nation situated 11.00°N and 61.00°W. The population which consists of 1.5 million people comprises approximately 300,000 households [1]. There are no published reports on the size of the dog population or the number of pets per household. During the late 1990's, there was considerable press coverage on dog attacks mainly highlighting fatal or near fatal attacks by pit bull terriers and their crosses [2,3]. This prompted the government to create a dangerous dogs act [4]aimed at banning certain breeds of dangerous dog and making owners liable if an animal has caused injury to persons. Trinidad and Tobago however, has no epidemiological data on the number of dog bites occurring per year and fatalities as a result of dog attacks are not recorded by the central statistical office.

The dog is known as man's best friend and it is estimated that over 52 million dogs cohabit with humans in the Unites States and the Center for Disease Control estimated that 799,700 out of 4.7 million bites occurring in 1994 required medical treatment [5]. The proportion of dog bites that are reported to authorities is variable with published estimates in the United States ranging from 10 – 50% [6]. Studies in developed countries have identified children under the age of 18 as the most frequent recipients of dog bites [6-8] with the ages that are most affected, varying among studies from, infants less than 1 year [9] to children aged 6 – 10 [10].

In addition to the severe physical trauma and potentially permanent disfiguring wounds sustained by a dog attack, dog bite victims are often burdened with emotional and psychological trauma [11]. Bites, no matter how severe, are a potential source of zoonotic infections particularly rabies and source of entry of pyogenic organisms and *Clostridium tetani *the cause of tetanus [12].

This study targets cases of dog bites using a questionnaire and informal interview to determine the potential magnitude of the dog bite risk to a vulnerable group in Trinidad. The survey records dog bites to children between the ages of 8–12 years attending primary schools in Trinidad to determine the risk factors associated with dog bites and to identify preventive strategies that could be implemented in order to reduce the incidence of injury to children from dog bites. This approach is intended to record factors associated with a bite incident through semi-structured interviews with children to record events which may otherwise go unreported. In addition, the semi-structured interview approach may prove valuable in empowering the target population to disclose information on associated risks factors which is often not ordinarily available to researchers and policy makers.

## Methods

The study was conducted in accordance with the guidelines of the Helsinki Declaration. Data for this study was gathered using a questionnaire, and formal ethical approval from the university ethics committee was not sought as no research other than information gathering was carried out on human subjects. The questionnaire was reviewed by the authors, teachers and/or parents, school principals and students before proceeding. The aims and objectives of the study were explained and students were asked if they were willing to participate and verbal informed consent was given by teachers and/or parents, school principals and students. Some students declined participating in the study.

### Schools studied and sample size determination

A list of primary schools in Trinidad and number of students enrolled for the year 2002 was obtained from the Ministry of Education, (Ministry of Education, Alexandria St, St. Clair POS, Trinidad). Out of a student population of 110 226 [1], a total of 1199 students were interviewed; however, 1109 students met the inclusion criteria of age 8–12 years. From estimates on dog bites in children based on previous studies, [6,13] the minimum number of students to be sampled was calculated based on an estimated prevalence (p) of nonfatal bites of 30% and an estimated error (L) of 3% at 95% confidence (z_α _= 1.96).

### Sampling procedure

A multistage sampling procedure was used. All educational districts were surveyed and the number of students interviewed from each district was proportional to the student population of the area. The number of schools from each district was selected proportionally based on the number of schools in the district and the student population of the area. Schools were chosen randomly whereby every fifth school on the data base was selected and permission to participate in the study sought from the principal.

### Administration of questionnaire

Verbal informed consent was granted by school principals to interview students individually. Students were also given the opportunity to accept or decline participating in the study. All students interviewed were selected randomly by their teachers and the questionnaire was administered by the first author. Students were also given the opportunity to talk freely on any subject related to dogs.

### Questionnaire

A bite was defined as an incident resulting in the breaking of the skin caused by the animal's teeth. None of the students interviewed were reluctant to discuss animal bites. Information about access of dogs to the school, number of dogs owned and whether or not they liked dogs were elicited first. Questions concerning dog bites were asked last. Students were asked if they were bitten by a dog between 2000 – 2003. If the year that they were bitten was not recalled, students were asked their age when bitten. In cases where a student was bitten more than once during the time period only the most recent event was recorded. Other information collected from the survey included the size of the dog (small, medium, large) involved in the incident, the part of the student's body injured, ownership of the dog, circumstances surrounding the attack, time of day and whether medical care was sought by the victim and who provided such care. Students were then given the opportunity to express their opinion on any thing they wished. No information was collected on the severity of the bite nor the breed of dog involved in the attack.

### Statistical Analysis

The risk factors associated with being bitten such as age, gender and location were tested for significance using the chi square test for independence. The independent sample t-test was used to determine if there was a significant difference between the mean age of being bitten between genders. To determine if there was any age-gender interaction, the Breslow-Day chi square for homogeneity of strata was calculated. To control for differences of odds ratios for being bitten, among age groups for males and females the Mantel-Haenszel adjusted odds ratio was calculated. Data analyses were done using the software statistical package for social sciences (SPSS) version 10 and Win Episcope 2.

## Results

### Demography of study population

This study interviewed 1199 students however 1109 met the inclusion criterion of belonging to the age group 8–12 years. In the study population, 688 (62.0%) students attended schools in urban areas and 421 (38.0%) in rural areas. Out of this group, 586 (52.8%) were girls and 523 (47.2%) were boys. Of the 1109, 290 (73.6%) students claimed owning a dog. Two hundred and ninety (26.1%) households kept no dogs while 153 (13.8%) had 4 or more dogs (Table 1). Students aged 10 years (29.8%) and females (31.2%) were most frequent in the sample population.

**Table 1:** Frequency (%) of the number of dogs owned per household

**Number of dogs present per household**	**No. (%) of owners**^a^
No dogs	290 (26.1)
1 dog	336 (30.3)
2 dogs	200 (18.0)
3 dogs	127 (11.5)
4 or more dogs	153 (13.8)
No response	3 (0.3)

^a ^Of a total of 1109 owners

### Age and gender

**Figure 1:**
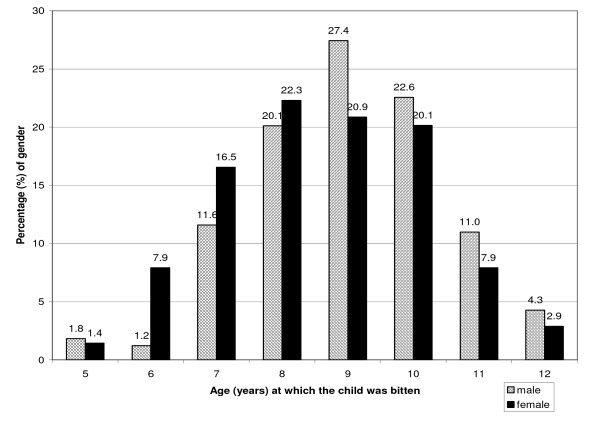
Age and gender distribution of victims of dog attack.

The age and gender distribution of bite victims is illustrated in Figure 1. Overall, (312) 28.1% of students reported being bitten by a dog during the period 2000–2003. Of the total study population, 170 (32.5%) boys and 142 (24.2%) girls interviewed were bitten. This difference was statistically significant. (χ^2 ^1 *df*, 95% CI, p = 0.002). There was no age-gender interaction, as the Breslow-Day chi square for homogeneity of strata was not significant. The Mantel-Haenszel adjusted odds ratio for males versus females, was 1.49, 95% C.I (1.15–1.94). The average age of being bitten was 9.0 years for boys and 8.5 years for girls. This difference in mean age was statistically significant between genders using the independent t-test, (p = 0.013 for 301 *df*). The modal age for boys and girls was 9 and 8 years respectively.

### Ownership of dog involved in the attack

The majority (263, 86.5%) of victims, were bitten by a dog known to them. This was either their family pet, (97, 31.9%) or a dog known to them but not owned by them (166, 54.6%). Unknown dogs however, were responsible for (41), 13.5% of bites. Owning a dog was also significantly associated with being bitten {χ^2 ^1 *df*, 95% CI, p = 0.003, OR = 1.57, 95% C.I (1.16–2.13)}

### Medical care

For this study, an indication of severity was being taken to a physician or primary care facility for treatment. Of those who were bitten, 234 (75.0%) said that some sort of medical care was needed (Table 2). From this group, 80 (34.2%) were treated at a primary care facility and 138 (59.0%) were treated by a relative, friend or treatment was self administered. Twenty five percent, (78) of children who were bitten reported that the wound was not given any form of treatment.

**Table 2:** Source of medical care to victim

**Source of Medical Care**^a^	**No. (%)**^b^
Private doctor/clinic	18 (7.7)
District Hospital	38 (16.2)
Health Centre	24 (10.3)
Relative	107 (45.7)
Friend	16 (6.8)
Self	15 (6.4)
Pet's owner (not relative or friend)	7(3.0)

^a ^9 (4.2%) could not remember who provided care

^b ^Of a total of 234 students who sought medical care

### Part of body bitten and frequency of bites

Bites to the lower leg, foot or toe were most often received by victims as shown in Table 3. Bites to these areas were received by 120 (38.5%) victims, 66 (40.2%) boys and 54 (38.6%) girls. The difference between genders was not statistically significant (p = 0.55). The majority of children 242 (77.6%) received one bite during the attack. One child reported receiving 15 bites; (these consisted of numerous cuts), the scars of which were shown to the first author.

**Table 3:** Frequency distribution of bites according to location of injury to the body^a^

**Location of bite**	**No. (%) bitten**
Lower leg, foot, toe	120 (39.3)
Hand, finger	68 (22.3)
Hip, buttock, thigh, knee	59 (19.3)
Head, face, neck	26 (8.5)
Forearm(below elbow), wrist	16 (5.2)
Shoulder, upper arm (above elbow)	9 (3.0)
Back, chest, trunk (including genitalia)	7 (2.3)

^a ^Eight students gave no response

### Month and time of the attack

Most children (183, 58.7%), could not recall the month that they were bitten, however, 243 (77.9%) were able to recall if they were bitten in the morning or the afternoon. Of the 243 students who recalled this information, 56 (17.9%) and 187 (59.9%) said they were bitten in the morning and afternoon respectively. One hundred and twenty-nine victims recalled the month over 2000 – 2003 that they were bitten. During this period 18 (14.0%) students said that they were bitten in August and 15 (11.6%) reported being bitten in April. The bite frequency rose sharply in April, July and August. The frequency peak occurred in April for those bitten in 2000, 2001 and 2003. The other bite frequency peaks were observed over the July and August months for each year of the study period.

### Circumstances leading to the dog attack

Many dog bites (103, 33.0%) were sustained without any interaction with the dog followed by playing or petting the dog (102, 32.7%), (Table 4). Only 23 (7.2%) reported being bitten as a result of teasing or provoking the dog. Unfortunately one student was bitten as a result of the dog's owner commanding it to attack.

**Table 4:** Frequency distribution of the circumstances and factors contributing to injury

**Circumstance surrounding the attack**	**No. (%) of victims reporting**
No interaction	103 (33.0)
Playing or Petting	102 (32.7)
Unspecified^a^	47 (15.0)
Teasing or Provoking	23 (7.4)
Dog with pups	13 (4.2)
Disciplining	9 (2.9)
Victim hurt dog	7 (2.2)
Ordinary interaction^b^	7 (2.2)
Dog commanded to attack	1 (0.3)

^a ^Students were unable to recall how the event occurred

^b ^Interaction other than playing or petting, includes feeding, grooming, bathing, walking the dog

## Discussion

This survey recorded remembered bites over a 4-year period and although a bite was well defined to each respondent, it is expected that perceptual and recall problems were a source of bias. Young children were often not likely to remember details of the bite incident. The overall bite frequency should therefore be taken as an underestimate. The results also show that being bitten by a dog is a common occurrence in children within the 7 to10 year age group. Similar patterns were observed by Beck et al., [13] from a study in the USA where children belonging to the 7 to 12 age group were the most frequent victims of unreported bites.

Only 34.2% of victims who treated their wounds, used the services of a health care facility, and 25.0% of all victims did not apply basic first aid care to their wound. Those who were not treated by a doctor or at a primary care facility, preferred to seek treatment from a relative or friend. This may be due to several factors, including the victim, his or her parent or guardian, not perceiving the bite wound to be severe enough to seek professional assistance. No information was gathered as to the severity of the wound sustained or the length of time for healing. As dog bites are highly contaminated with several bacterial pathogens [12], bite wounds, regardless of severity, serve as a potential source of entry of anaerobic bacteria, particularly *Clostridium tetani *[12] and other bacteria such as *Staphylococcus sp*, *Streptococcus sp*, *Pastuerella multocida *and *Capnocytophaga canimorsus *[14]. It is therefore important that all children should receive prophylactic treatment for potential infections after a bite incident. Fortunately, the immunization system in Trinidad is well structured and most children have been immunized against tetanus by age 10 years. This factor may influence the apparent lackadaisical response to tending to bites received. Worldwide, carnivores, including the dog are important vectors for rabies, however in the Americas, bats are also major vectors[15]. Therefore, another factor which may influence attitudes toward seeking prompt medical care is the apparent absence of rabies in the canine population in Trinidad. The vampire bat as a vector for bovine rabies in Trinidad and a potential source of human rabies has been established since 1936 [16], however, the canine population is considered rabies-free. The public's knowledge of other potentially harmful zoonotic diseases from dog bites may therefore be quite limited.

This study showed that there was a significant association between the victims' gender and the likelihood of being bitten, with boys being 1.49 more times likely than girls to be bitten. Male victims predominated at ages 5 and 9 and above, however, female victims outnumbered males in the 6 to 8 age group. A review of data on dog bites in the United States showed that males were bitten significantly more often than females across all age groups [6]. A similar study in Canada from data taken from 16 hospitals indicated that 57.9% of all injuries related to dog bites were to males [17].

The severity of the sequelae to bite wounds was not assessed in this study, however the interviewer noted that many children were severely traumatized and some bite wounds received were highly disfiguring, to limbs and face. Two boys included in this study reported being bitten on their genitals and one boy who was not included in the study population had severely disfiguring injuries to his face which were received when he was bitten as an infant. Studies conducted elsewhere, have indicated that functional and aesthetic consequences are estimated to occur in 1 – 3% of all bites [12,17-19]and child victims of dog bites should also be considered at risk of developing psychological injuries such as post traumatic stress disorder [11]. The vast majority of the biting dogs (86.5%) were owned animals and known to the victim. This is in agreement with other published data [6,17,20]. As there is no formal licensure for dogs in Trinidad, those classified as known have some sort of referral household which was recognized by the child. Unknown dogs were those which were not recognized by the child. Even though there is a very large stray dog population in Trinidad, dogs classified as unknown to the victim accounted for only 13% of bites, suggesting that unknown roaming dogs, though posing a risk of injury, are not the most significant source of dog bites to children in the country. It must be noted however, that it is very difficult to accurately classify dogs in Trinidad as many roaming animals have owners or some sort of referral household.

Most studies on dog injuries are derived from hospital data, often from emergency departments. In published reports of studies derived from hospital data [21-23], the most common site of injury by dog bites were to the face, head and neck, especially in children under 5 years. In our study, children under 8 years were not interviewed and we found that the lower leg, foot or toe were the most frequent site of bite wounds (39.3%) and bites to the face accounted for only 8.5% of all wounds. However, in a review of hospital records of animal bites in Thailand, bites to the upper extremities were more frequent in children under 6 years with the trend decreasing with increasing age, with attacks to the lower extremities becoming more common [24]. The relationship between the animal's head and the height of the victim are known indicators with respect to the site of injury, and young children therefore, are bitten more often on their face, head and upper extremities. It is also expected that severe dog bites and those sustained to the face are more likely to require medical attention as there is potential to result in permanent physical aesthetic damage to the child. To the contrary, in our study only 5 of the children who reported receiving bites to the head, face or neck sought professional medical care. It is also pertinent to mention that in our study, bites which most often received medical attention were those inflicted on the lower leg, foot or toe. The importance of considering the sources of data when comparing studies can therefore not be overemphasized.

A high proportion of children who were able to recall the month that they were bitten, indicated that the event occurred in either April or August. For the period 2000 – 2003, there were fewer bites occurring between September and December and except for 2000, between January and April. This trend may be explained in part by the fact that the month of April and the July/August period are the months when children are on vacation from school, often not well supervised and are more likely to have some sort of interaction with a biting dog which was known to them but not necessarily owned by them. These findings are in agreement with other studies which indicate that the peak incidence of bites occurred during the summer months or when children were not in school [17,20,24]. Many (33.0%) attacks in our study were unprovoked. Children, who were attacked without any prior interaction with the dog, reported either walking, running, or on their bike when attacked. Reports by others have also indicated that almost 30 – 50% of attacks to young children are unprovoked [17,25]. Other victims, in this study, reported invading the dog's territory such as passing close to a dog or touching a dog with pups, feeding or disciplining a dog or parting fights. Close supervision of children when caring for dogs is therefore important in reducing risk of injury.

## Conclusion

Our findings in this study have demonstrated that primary school children in Trinidad are bitten by dogs under very common circumstances but a frequency of 28% of dog bites in the 8–12 year age group is high enough to warrant the need for educating children and adults on responsible pet ownership. As 87% of those bitten were attacked by a dog known to them it is imperative that dog owners be aware of their responsibility in safe guarding the public from injury. In addition, the following measures are recommended:

(i). Animal social behaviour should also be taught to children so that aggression as a result of a pet displaying territorial behaviour may be avoided. (ii). Potential pet owners should also be informed by pet shop proprietors, dog breeders or the humane society of the type of pet best suited to a household so that young children may not be injured. (iii). The Dangerous dogs act 2000 (Table 5) is yet to be put into law and it is hoped that once it is part of the legislature it would encourage all dog owners to be more responsible. (iv). Law enforcement officials would have to collaborate with other agencies such as the humane society and kennel clubs in order to increase public awareness of the consequence of not exercising responsible pet ownership. (v). One of the major constraints to enforcing the legislature is the number of feral dogs and those with referral households which evidently are allow to roam. It is therefore imperative that the city and county councils facilitate prompt removal of roaming/stray dogs from the streets and their disposal humanely. The humane society and other non-governmental animal welfare networks would therefore play a more active role in this regard.

**Table 5:** Excerpts from The Dangerous Dogs Act, 2000 [4]

Dangerous dogs are those defined as dogs or crosses of the pit bull terrier, Fila Brasileiro, Japanese Tosa are not to be imported
These dogs must be spayed or neutered, and are not to be bred
All owners of the above breeds must register and insure their dogs
All dangerous dogs must be identified
Persons must keep the dog under proper control on private premises. These premises must be secured to prevent the escape of the dog. An owner who contravenes this is liable to a fine of 50,000.00 TTD^a ^and imprisonment for one year
If a dangerous dog injures a person the owner is liable to a fine of 100000 TTD and imprisonment for one year.
Where a dangerous dog kills a person or causes the death of a person the owner or keeper of the dog is liable to a fine of 200,000 TTD and imprisonment for 10 years
Dogs of any type other than dangerous dogs that present a danger to the public are also included in the act

^a ^1 USD = 6.30 TTD January 2008

## Competing interests

The author(s) declare that they have no competing interests.

## Authors' contributions

KG designed and executed the study and analyzed data. A. Adesiyun contributed to study design and edited the manuscript. Both author's have read and approved the final manuscript.

## Pre-publication history

The pre-publication history for this paper can be accessed here:


